# School-Based Universal Mental Health Prevention Programs for Children Aged 6 to 9 Years in Low- and Middle-Income Countries: Protocol for a Scoping Review

**DOI:** 10.2196/87105

**Published:** 2026-04-10

**Authors:** Cherith Langenhoven-Heber, Katherine Sorsdahl, Emily Garman, Claire van der Westhuizen

**Affiliations:** 1 Department of Psychiatry and Mental Health, Alan J Flisher Centre for Public Mental Health, University of Cape Town, 46 Sawkins Road, Rondebosch, Cape Town, Western Cape, 7700, South Africa, 27 21 406 6663

**Keywords:** mental health, well-being, child mental health, school based, universal prevention, prevention, mental health promotion, children, low- and middle-income countries, LMICs

## Abstract

**Background:**

While the onset of most mental health conditions occurs in later childhood or adolescence, the early years present an important window for prevention. Worldwide, universal school-based prevention interventions have been shown to be effective in promoting child mental health. Nevertheless, little is known about universal school-based prevention programs targeting children aged 6 to 9 years in low- and middle-income countries (LMICs).

**Objective:**

This scoping review aims to identify promising, potentially feasible, and possibly evidence-based school-based universal prevention mental health programs for children aged 6 to 9 years in LMICs.

**Methods:**

This scoping review is planned according to the Joanna Briggs Institute framework developed by Arksey and O’Malley, with enhancements proposed by Levac et al. Eligible studies will focus on universal prevention school-based mental health interventions with feasibility and/or mental health outcomes that include children aged 6 to 9 years. Additional inclusion criteria are peer-reviewed published and unpublished studies, including gray literature such as theses and dissertations, preprints, conference proceedings and papers, and government reports and publications. There are no limits to the date of publication, but only studies published in English and conducted in LMICs will be included. The search strategy was iteratively developed and finalized, following which major databases, namely, PubMed (MEDLINE), Scopus (Embase), EBSCOhost (PsycInfo, CINAHL, PsycArticles, ERIC, Teacher Reference Center, Academic Search Premier, and SocINDEX), CENTRAL, ProQuest, the Open Science Framework, and the International Clinical Trials Registry Platform, were searched. Titles and abstracts are being screened in addition to manual searches of the reference lists of previous reviews. Experts will be consulted for further study suggestions, and reference mining will be conducted with the included studies. A descriptive and numerical summary will reflect basic program characteristics. A preliminary data extraction chart curated around the research questions will be used for deductive and inductive data analysis. The entire scoping review will be reported according to the PRISMA-ScR (Preferred Reporting Items for Systematic Reviews and Meta-Analyses extension for Scoping Reviews) guidelines.

**Results:**

In November 2025, the search strategy was finalized. All database searches were completed by early December 2025, yielding 7511 records. After removing duplicates (n=3022), 4489 records underwent title and abstract screening. Of these, 25 records met the criteria for full-text review, which is currently ongoing. Data extraction and analysis should take place between April and May 2026. The scoping review findings will be submitted to a relevant peer-reviewed journal by July 2026 and presented at conferences.

**Conclusions:**

This review will provide a description of feasible and potentially effective school-based universal mental health programs specific to children aged 6 to 9 years living in LMICs. The findings may assist with efficient insight into available programs for implementation or adaptation among the stipulated population in the future.

## Introduction

Mental health conditions are highly prevalent in low- and middle-income countries (LMICs), with most conditions emerging early in life. It is estimated that 82% of people with mental health conditions live in LMICs [1]. While most mental health conditions present prior to adulthood [2], worldwide, approximately 34% of people living with mental health conditions have an age of onset younger than 14 years [3]. Furthermore, the global median reported age of onset is 7 to 9 years for attention-deficit/hyperactivity disorder, 7 to 15 years for oppositional defiant disorder, and 9 to 14 years for conduct disorder [4].

In LMICs, although mental health conditions are highly prevalent, up to 90% of those affected receive no care—a disparity known as the “treatment gap” [5,6]. Low political interest, stigma associated with mental health conditions, lack of appropriately trained staff [7], and poor funding for mental health care resulting in insufficient availability and implementation of mental health services [8] have been identified as contributors to the treatment gap in LMICs. This is evident especially for youth in LMICs, who are reported to have a range of 0.05 to 0.12 child and adolescent mental health workers per 100,000 youth [8]. Therefore, the prevalence of mental health conditions, age of onset, and high treatment gap for youth highlighted motivate a focus on mental health prevention interventions for children in LMICs.

The World Health Organization [8], within its Comprehensive Mental Health Action Plan, strongly recommended that interventions should begin in early childhood through universal approaches to prevent poor mental health. Furthermore, worldwide, research evidence has shown that school-based prevention programs are effective in promoting youth mental health, whereas universal school-based programs are especially considered a cost-effective and feasible approach at the population level [7]. More specifically, universal school-based programs have been effective in preventing mental health problems in children as young as 2 years of age [9-12]. Indeed, research indicates that universal social and emotional learning [9,10], resilience [11], and self-regulation [12] school-based programs have significantly improved behaviors, school climate, social skills, emotional skills, and academic abilities and reduced psychological distress, substance use, and school suspensions in youth. Additionally, one meta-analysis conducted by Hayes et al [13] reported that universal prevention school-based programs were effective in preventing depression and anxiety in youth aged 8 to 18 years.

In LMICs, schools offer an opportunity for sustainable mental health promotion and prevention programs among youth [14,15] provided that programs are culturally relevant [16]. One scoping review and one systematic review synthesized evidence primarily on the effectiveness of any universal school-based mental health programs being implemented among children and adolescents in LMICs [16,17]. Bradshaw et al [17] obtained inconclusive results, whereas the scoping review by Harte and Barry [16] supported the efficacy of universal programs among children and adolescents aged 5 to 19 years. Despite a growing evidence base of effective universal school-based programs promoting child mental health, it is still unclear whether such programs are feasible for children aged 6 to 9 years living in LMICs. Indeed, some reviews of the literature have been either too broad in terms of age [13,16,17] or synthesized evidence across mixed prevention approaches (universal, indicated, and selective) [16]. Others have only included randomized controlled trials and/or quasi-randomized controlled trials with specific mental health outcome measures such as anxiety and depression symptoms [13,17,18].

To fill the research gap, we propose to conduct a scoping review to identify and summarize universal school-based mental health programs designed for children aged 6 to 9 years living in LMICs that have demonstrated (1) promising outcomes in feasibility and acceptability or (2) an improvement in well-being and mental health, typically shown through psychosocial measures.

## Methods

### Overview

The scoping review will be conducted according to the Joanna Briggs Institute (JBI) methodology, developed to guide rigorous knowledge synthesis along with trustworthy and transparent research practice [19]. The JBI scoping review framework, developed by Arksey and O’Malley [20] with enhancements proposed by Levac et al [21], will be used. This framework includes six stages for conducting scoping reviews: (1) identifying the research question, (2) identifying relevant studies, (3) study selection, (4) charting the data, (5) collating findings and summarizing and reporting the results, and (6) stakeholder consultation. As required by the JBI framework, the protocol is reported according to the PRISMA-ScR (Preferred Reporting Items for Systematic Reviews and Meta-Analyses extension for Scoping Reviews) checklist [22] to ensure complete reporting of the scoping review process.

### Stage 1: Identifying the Research Question

The scoping review aims to address the following research question: what potentially feasible school-based universal mental health programs are available for children aged 6 to 9 years in LMICs? However, this research question may be further refined if necessary throughout the scoping review process. Secondary research questions are:

1. What components were included in these interventions? Which components were evidence based?
2. Were these interventions developed or adapted for use in their specific context?
3. Were these interventions efficacious, feasible, and acceptable?
4. Which of these interventions reflects a sustainable approach?

### Stage 2: Identifying Relevant Studies

#### Overview

On the basis of the research question, a comprehensive yet feasible search strategy must be developed with clear inclusion and exclusion criteria, including the type of sources searched, as recommended by the JBI [19,21]. The authors have developed inclusion and exclusion criteria for the scoping review. To retrieve a full range of school-based universal mental health programs for children, all English-language peer-reviewed published and unpublished articles, along with full-text studies from other sources, will be included. These eligibility criteria are delineated and detailed further below.

#### Eligibility Criteria

There are no limits to the date of publication, but the intervention must have been conducted in LMICs. Due to concerns regarding the dearth of programs available for the stipulated population in LMICs, the authors chose to refrain from applying any limits to the date of publication. All peer-reviewed full-text studies, including nonjournal articles, conference proceedings and papers, and government reports and publications published in English, will be included. The studies must focus on universal prevention school-based mental health interventions and include children aged 6 to 9 years. Further details are provided in Table 1.

**Table 1.** Eligibility criteria.

Category	Inclusion criteria	Exclusion criteria
Language	Studies published in English	Studies published in a language other than English
Target population	Studies that include children aged 6 to 9 y at the time of recruitment	Studies that only focus on younger children aged 0 to 5 y or that only include participants aged 10 y and above
Interventions	Studies that investigate universal prevention school-based mental health interventions	Studies that investigate interventions not focused on universal prevention (eg, selective, indicated, or treatment intervention or program studies)
Geographic location	Studies conducted in LMICs^a^	Studies conducted in HICs^b^
Literature sources	Primary studies; secondary studies published in peer-reviewed journals or unpublished; and gray literature, including theses and dissertations and intervention-related literature that appears in conference proceedings and papers and government reports and publications	Gray literature outside of conference proceedings and papers and government reports and publications
Study design	Any study design that includes an intervention	Study designs without an intervention
Outcome measures	Studies with feasibility and/or mental health outcomes, which include the use of psychosocial measures	Studies solely focused on prevalence and/or associations and studies that do not measure any feasibility outcomes or include mental health outcome measures

a LMIC: low- and middle-income country.

b HIC: high-income country.

#### Search Strategy

As recommended by the JBI [19], stage 2 also involves developing a comprehensive search strategy. A trial search using 2 databases, namely, EBSCOhost and Scopus, was conducted using preliminary search terms, including MeSH (Medical Subject Headings) terms. This was achieved with the assistance of an institutional librarian from the health science faculty library research department of the University of Cape Town. After analyzing titles, abstracts, and index words of the articles retrieved in the trial search, the key search terms were revised to ensure that the search strategy culminates in articles relevant to this review. Table 2 shows the final search terms.

**Table 2.** Search strategy terms.

Keyword	Search terms^a^
Mental health	“Mental Health” OR “Wellbeing” OR “Emotional Regulation” OR “Resilience” OR “Self-esteem” OR “Coping Skills” OR “Self-regulation” OR “Socio-emotional Characteristics” OR “Socio-emotional Skills” "Mental Health"[Mesh] OR “Wellbeing” OR "Child Health"[Mesh] OR "Emotional Regulation"[Mesh] OR “Resilience” OR "Resilience, Psychological"[Mesh] OR “Self-esteem” OR "Self-concept"[Mesh] OR "Coping Skills" OR “Self-regulation” OR "Self-control"[Mesh] OR “Socio-emotional Characteristics” OR “Socio-emotional Skills”
Program	“Program*” OR “Promotion” OR “Life skills” OR “Socio-emotional Learning” OR “Intervention” OR “Psycho-education” “Program*” OR “promotion” OR “Life Skills” OR “Socio-emotional learning” OR “Intervention” OR "Preventive Health Services"[Mesh] OR "Psychosocial Intervention"[Mesh] OR “Psycho-education”
Children	“Child*” OR “Primary School*” OR “School Child*” OR “Elementary School*” "Child"[Mesh] OR “Primary School*” OR “Elementary school*”
School based	“School-based” OR “School based” OR “School Setting” OR “Curriculum” OR “School” “School-based” OR “School based” OR “School setting” OR "Curriculum" OR “School” OR “School Mental Health Services"[Mesh] OR "School Health Services"[Mesh]
Universal	“Universal” OR “Group-based” OR “Group based” OR “Classroom-based” OR “Classroom based” OR “Classroom” “Universal” OR “Group-based” OR “Group based” OR “Classroom-based” OR “Classroom based” OR “Classroom”
LMICs^b^	“Deprived Countries” OR "Deprived Population" OR "Deprived Populations" OR "Developing Countries" OR "Developing Country" OR "Developing Economies" OR "Developing Economy" OR "Developing Nation" OR "Developing Nations" OR "Developing Population" OR "Developing Populations" OR "Developing World" OR "LAMI Countries" OR "LAMI Country" OR "Less Developed Countries" OR "Less Developed Country" OR "Less Developed Economies" OR "Less Developed Nation" OR "Less Developed Nations" OR "Less Developed World" OR "Lesser Developed Countries" OR "Lesser Developed Nations" OR LMIC OR LMICS OR Low GDP OR "Low GNP" OR "Low Gross Domestic" OR "Low Gross National" OR "Low Income Countries" OR "Low Income Country" OR "Low Income Economies" OR "Low Income Economy" OR "Low Income Nations" OR "Low Income Population" OR "Low Income Populations" OR "Lower GDP" OR "lower gross domestic" OR "Lower Income Countries" OR "Lower Income Country" OR "Lower Income Nations" OR "Lower Income Population" OR "Lower Income Populations" OR "Middle Income Countries" OR "Middle Income Country" OR "Middle Income Economies" OR "Middle Income Nation" OR "Middle Income Nations" OR "Middle Income Population" OR "Middle Income Populations" OR "Poor Countries" OR "Poor Country" OR "Poor Economies" OR "Poor Economy" OR "Poor Nation" OR "Poor Nations" OR "Poor Population" OR "Poor Populations" OR "poor world" OR "Poorer Countries" OR "Poorer Economies" OR "Poorer Economy" OR "Poorer Nations" OR "Poorer Population" OR "Poorer Populations" OR "Third World" OR "Transitional Countries" OR "Transitional Country" OR "Transitional Economies" OR "Transitional Economy" OR "Under Developed Countries" OR "Under Developed Country" OR "under developed nations" OR "Under Developed World" OR "Under Served Population" OR "Under Served Populations" OR "Underdeveloped Countries" OR "Underdeveloped Country" OR "underdeveloped economies" OR "underdeveloped nations" OR "underdeveloped population" OR "Underdeveloped World" OR "Underserved Countries" OR "Underserved Nations" OR "Underserved Population" OR "Underserved Populations" OR Afghanistan OR Albania OR Algeria OR “American Samoa” OR Angola OR Armenia OR Azerbaijan OR Bangladesh OR Belarus OR Byelarus OR Belorussia OR Belize OR Benin OR Bhutan OR Bolivia OR Bosnia OR Botswana OR Brazil OR Bulgaria OR Burma OR “Burkina Faso” OR Burundi OR “Cabo Verde” OR “Cape Verde” OR Cambodia OR Cameroon OR “Central African Republic” OR Chad OR China OR Colombia OR Comoros OR Comores OR Comoro OR Congo OR “Costa Rica” OR “Côte d'Ivoire” OR Cuba OR “Democratic People’s Republic of Korea” OR Djibouti OR Dominica OR “Dominican Republic” OR Ecuador OR Egypt OR “El Salvador” OR Eritrea OR Ethiopia OR “Equatorial Guinea” OR Fiji OR Gabon OR Gambia OR Gaza OR “Georgia Republic” OR Georgia OR Ghana OR Grenada OR Grenadines OR Guatemala OR Guinea OR “Guinea Bissau” OR Guyana OR Haiti OR Herzegovina OR Hercegovina OR Honduras OR India OR Indonesia OR Iran OR Iraq OR “Ivory Coast” OR Jamaica OR Jordan OR Kazakhstan OR Kenya OR Kiribati OR Korea OR Kosovo OR Kyrgyz OR Kirghizia OR Kirghiz OR Kyrgyzstan OR “Lao PDR” OR Laos OR Lebanon OR Lesotho OR Liberia OR Libya OR Macedonia OR Madagascar OR Malawi OR Malay OR Malaya OR Malaysia OR Maldives OR Mali OR “Marshall Islands” OR Mauritania OR Mauritius OR Mexico OR Micronesia OR Moldova OR Mongolia OR Montenegro OR Morocco OR Mozambique OR Myanmar OR Namibia OR Nepal OR Nicaragua OR Niger OR Nigeria OR Pakistan OR Palau OR “Papua New Guinea” OR Paraguay OR Peru OR Philippines OR Principe OR Romania OR Rwanda OR Ruanda OR Samoa OR “Sao Tome” OR Senegal OR Serbia OR “Sierra Leone” OR “Solomon Islands” OR Somalia OR “South Africa” OR “South Sudan” OR “Sri Lanka” OR “St Lucia” OR “St Vincent” OR Sudan OR Surinam OR Suriname OR Swaziland OR Syria OR “Syrian Arab Republic” OR Tajikistan OR Tadzhikistan OR Tajikistan OR Tadzhik OR Tanzania OR Thailand OR Timor OR Togo OR Tonga OR Tunisia OR Turkey OR Turkmen OR Turkmenistan OR Tuvalu OR Uganda OR Ukraine OR Uzbek OR Uzbekistan OR Vanuatu OR Venezuela OR Vietnam OR “West Bank” OR Yemen OR Zambia OR Zimbabwe OR “low- and middle-income”

a For each keyword, the first bullet point represents the generic search string applied across databases, while the second point includes database-specific MeSH (Medical Subject Headings) terms used exclusively for PubMed searches.

b LMIC: low- and middle-income country.

Next, a systematic search incorporating the search terms was performed across all selected databases [19] using various search processes and query strings (Multimedia Appendix 1). PubMed (MEDLINE), Scopus (Embase), EBSCOhost (PsycInfo, CINAHL, PsycArticles, ERIC, Teacher Reference Center, Academic Search Premier, and SocINDEX), and CENTRAL were searched. Additionally, ProQuest, the Open Science Framework, and the International Clinical Trials Registry Platform were searched for gray literature, namely, conference papers, dissertations and theses, and preprints, as mentioned above. The full search was an iterative process as the authors first evaluated whether the full search culminated in sufficient representative articles. The authors decided to exclude the “LMICs” search term during the database search because too few results were found, as was done in a similar review by Bradshaw et al [17]. In addition, colleagues who are experts in the field of school-based mental health programs in LMICs will be consulted for applicable research literature recommendations in case any relevant literature was missed in the search. Moreover, the programs presented in other applicable reviews identified through the database search will be independently assessed by 2 reviewers for inclusion.

### Stage 3: Study Selection

This stage involves selecting the relevant articles for the scoping review by screening all the literature identified. Therefore, all the literature identified in stage 2 will be saved on EndNote (version 21; Clarivate Analytics) [23] and loaded onto Rayyan (Qatar Computing Research Institute), a web-based application and mobile app used to facilitate the selection of suitable studies [24]. There are 2 steps to study selection using the study inclusion and exclusion criteria as a guide. First, the study titles and abstracts will be screened independently by 2 reviewers to identify potentially relevant articles for full-text review [24]. Additionally, the review team will meet regularly to discuss any difficulties related to study selection, and this may result in revising the search strategy [21]. At this point, the ascendancy approach, which entails searching the reference list of each source to identify any applicable interventions, will be used with reviews and gray literature from the search results. Relevant studies identified will be added to the title and abstract screening phase for review. Second, the full text of each study will be assessed for eligibility, resulting in the final studies for inclusion in the scoping review [24].

At each step in the selection process, any disagreement on the inclusion or exclusion of literature sources may be resolved through consensus or the involvement of a third party [19]. Once the full-text study selection process is complete, the reviewers will use the ascendancy approach again, with the final included studies, to identify any final study additions to the scoping review. While a scoping review does not require the quality assessment of studies [19], we will apply the JBI critical appraisal tool to each included study. This will highlight the methodological quality of each study, which will be important for the interpretation of the study findings.

### Stage 4: Charting the Data

This stage begins with the development of a draft charting table with preliminary domains [21]. In addition to basic study characteristics, data will be extracted according to the study objective and research questions. For example, information will be charted on prior adaptation work and any feasibility testing. A Microsoft Excel spreadsheet will be used to input the extracted data from each study (Multimedia Appendix 2), and the domains will act as headings on the table. The domains currently included are (1) author, (2) publication year, (3) country, (4) setting, (5) target population, (6) sample size, (7) type of intervention, (8) program adaptations for the setting, (9) program components, (10) evidence on the components, (11) method of delivery and facilitator type, (12) duration of program and sessions, (13) outcomes (measures, feasibility, and acceptability), (14) sustainability indicators, and (15) research findings. In addition, a domain for the outcome of the JBI quality appraisal tool for each study will be listed to provide an overview of study rigor. Additional domains may be added after consultation with colleagues in the field of public mental health and during data analysis in the next stage. The table will be piloted independently by 2 reviewers to minimize bias and evaluate whether all the necessary information is extracted. The reviewers will perform an extraction and verification using a 10% sample of the included studies and amend the table if necessary. Once the table is finalized, data from the remaining studies will be extracted and added to the table by the first author.

### Stage 5: Collating Findings and Summarizing and Reporting the Results

**Figure 1.**
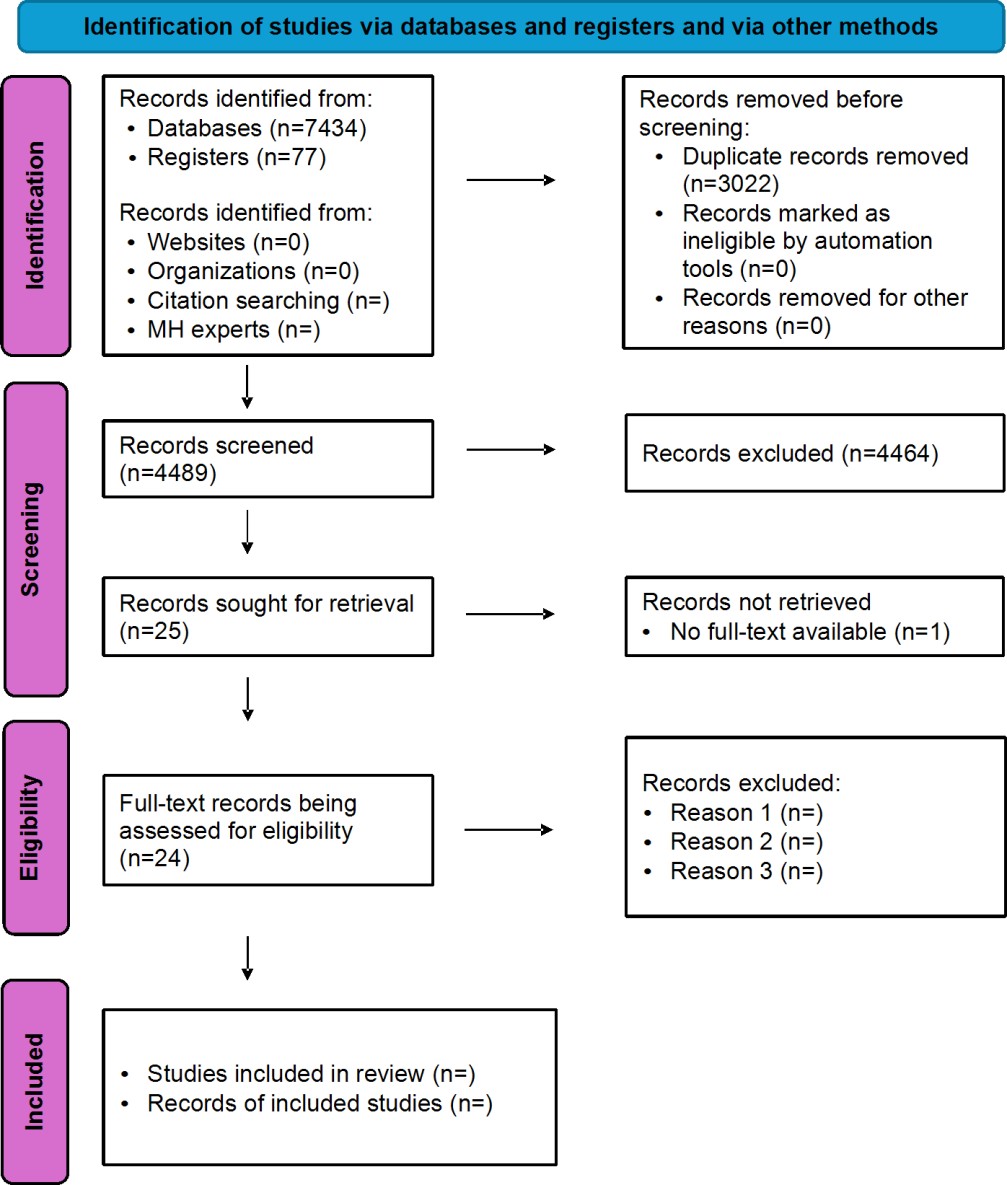
PRISMA (Preferred Reporting Items for Systematic Reviews and Meta-Analyses) 2020 flow diagram (adapted by the first author). MH: mental health.

The results of the study selection process from screening until final selection will be reported [21] according to the PRISMA-ScR guidelines [22], which include a narrative summary of the review selection process and outcomes [19]. Moreover, as required by the JBI framework, it will be accompanied by the PRISMA (Preferred Reporting Items for Systematic Reviews and Meta-Analyses) 2020 flow diagram (Figure 1) and tables or charts (Multimedia Appendix 2) used as visual tools to depict the scoping review process and selected mental health programs [19].

This stage also involves analyzing the data, reporting the results, and discussing the implications of the findings [21]. The first author will compile and present a descriptive and numerical summary of all the study characteristics, including the number of studies, type of study design, year of publication, population, type of intervention, and setting [20]. This stage will also include a thematic summary of the included programs [21] according to program content, outcome measures, method of delivery, duration of the program, type of facilitator, findings, and any other notable theme that may arise from the included studies. The data will be collated and described according to predetermined domains (see Multimedia Appendix 2 for the preliminary charting table). This deductive approach will enable description of the programs according to their components, evidence base, prior adaptations, feasibility, acceptability, and perceived sustainability, thus addressing the research questions. Information regarding other domains identified inductively will also be reported. Although this study is largely descriptive in nature, the collated data on the programs will be assessed for the level of resources and expertise required to deliver them. This initial assessment will inform aspects of the stakeholder consultations described in stage 6.

### Stage 6: Stakeholder Consultation

According to Levac et al [21], consultation is required throughout the scoping review to establish methodological rigor. Therefore, the entire review process will involve informal consultation with stakeholders such as Centre for Public Mental Health (CPMH) staff, research experts, study supervisors, and a librarian [19]. Thus far, a librarian has assisted with finalizing the search query string and selection of databases; CPMH staff members have provided guidance on databases for gray literature searches and will be involved in regular meetings (1-2 weekly) during the data analysis phase to guide the presentation and interpretation of the data. Experts working in the fields of education or child mental health identified through CPMH staff will be approached to suggest possible programs for inclusion in the review. In addition, they will be contacted to give input on the scoping review findings via email or face-to-face discussions.

### Ethical Considerations

As no human participants were involved in this research, no ethics approval is required.

## Results

Funding for this study has been ongoing since February 2024, as it is part of a larger study. In November 2025, the search strategy was finalized, and all database searches were completed by December 2, 2025. A pilot search indicated that including the LMIC-related query string greatly limited the search results. Instead, we opted to use this screening criterion at the title and abstract review. In December 2025, we proceeded with the first phase of screening, which includes removing duplicates and the title and abstract review. Currently, the search has yielded 7511 records; 3022 (40.23%) duplicates were removed, and 4489 (59.77%) records were screened at the title and abstract phase, which resulted in 25 (0.33%) studies ready for full-text review. We are in the process of full-text review, and data extraction and analysis will occur between April 2026 and May 2026. We anticipate the completion of the scoping review manuscript for submission by July 2026. Once published, these findings will be presented at a conference to contribute to academic literature and inform future research.

## Discussion

From the scoping review, we expect to identify programs that show some level of effectiveness, sustainability, or feasibility and acceptability suitable for children aged 6 to 9 years in LMICs. We know that the value of universal school-based programs is a highly contentious subject in academia currently, especially in LMICs [25,26]. Therefore, it is imperative that we find a program or components within these programs that show promise and may inform future work. Moreover, a detailed review protocol founded on evidence-based guidelines is essential for a well-executed scoping review, resulting in transparent and rigorous research practice [21]. Conducting a formal quality appraisal of each study contributes methodological depth that extends past conventional scoping review practices. This is important as there are various programs that have a distinct focus, such as social and emotional learning, resilience, and self-regulation, and it will be interesting to see the differences in study quality. Moreover, we might find that there is an overlap of components across the different types of programs and that perhaps a combination of these components could yield a more comprehensive program.

It was highlighted by Seekles et al [27] that many reviews have been conducted but lack focus on this specific age group, highlighting a critical gap in research reviews. This is evident by the large availability of adolescent mental health research, yet the age of 6 to 9 years is a highly important developmental stage for children. Two more recent reviews by Hayes et al [13] and Harte and Barry [16] have since included this age range but, unfortunately, focused on the effectiveness of programs, which provides less information on the feasibility and acceptability or sustainability of each program. This review will address this gap.

Apart from the future research contribution of this scoping review, there are additional strengths to its methodology. The application of the JBI framework ensures rigorous, trustworthy, and transparent research practice [21]. Additionally, adhering to the PRISMA-ScR guidelines [22] will ensure comprehensive reporting within the scoping review [28]. In contrast, limiting the geographical location to LMICs may result in the exclusion of potentially feasible school-based universal mental health programs conducted elsewhere. Furthermore, being limited to studies published in English may exclude studies conducted in numerous LMICs where other languages are spoken. The authors are also aware that the lack of limitation on the publication dates could result in the inclusion of outdated programs. However, while conducting the literature review, it was clear that children aged 6 to 9 years in LMICs are often ignored in research, which could already limit the number of programs identified. Additionally, we will be cautious when reporting the findings of outdated interventions. Prior to conducting the scoping review, an interim protocol has been uploaded and registered on the Open Science Framework platform [29], which will be updated iteratively. Furthermore, the findings of the scoping review will be submitted to a relevant academic journal and presented at one or more conferences to contribute to academic literature and inform future research.

## Supplementary material

10.2196/87105 Multimedia Appendix 1 Database search strategies.

10.2196/87105 Multimedia Appendix 2 Data extraction tool.

## Abbreviations

CPMH: Centre for Public Mental Health
JBI: Joanna Briggs Institute
LMIC: low- and middle-income country
MeSH: Medical Subject Headings
PRISMA: Preferred Reporting Items for Systematic Reviews and Meta-Analyses
PRISMA-ScR: Preferred Reporting Items for Systematic Reviews and Meta-Analyses extension for Scoping Reviews
