# Supplementary material for: School-Based Universal Mental Health Prevention Programs for Children Aged 6 to 9 Years in Low- and Middle-Income Countries: Protocol for a Scoping Review
Source: JMIR Res Protoc. 2026 Apr 10;15:e87105. doi: 10.2196/87105 (PMC13068186; doi:10.2196/87105)
Supplement: Multimedia Appendix 1 [file resprot-v15-e87105-s001.docx]

**Supplementary Database Search Material**

## Searched any year of publication until 2^nd^ December 2025 (applicable to every database)

## PubMed = 902 results (CL-H & CR)

Paste the following search string into the search query row and select **Title/Abstract:**

("Mental Health"[Mesh] OR “Wellbeing” OR "Child Health"[Mesh] OR "Emotional Regulation"[Mesh] OR “Resilience” OR "Resilience, Psychological"[Mesh] OR “Self-esteem” OR "Self-concept"[Mesh] OR "Coping Skills" OR “Self-regulation” OR "Self-control"[Mesh] OR “Socio-emotional Characteristics” OR “Socio-emotional Skills”) AND (“Program*” OR “Promotion” OR “Life Skills” OR “Socio-emotional learning” OR “Intervention” OR "Preventive Health Services"[Mesh] OR "Psychosocial Intervention"[Mesh] OR “Psycho-education”) AND ("Child"[Mesh] OR “Primary School*” OR “Elementary school*”) AND (“School-based” OR “School based” OR “School setting” OR "Curriculum" OR “School” OR “School Mental Health Services"[Mesh] OR "School Health Services"[Mesh]) AND (“Universal” OR “Group-based” OR “Group based” OR “Classroom-based” OR “Classroom based” OR “Classroom”)

## EBSCOHOST = 2,709 results (CL-H & CR) only downloaded 1829 onto Endnote (see* for explanation- 880 Duplicates were removed by EBSCHOST when downloading)

1. Paste the following whole search string into the search query in three separate rows
2. Select **Row 1 Title and Row 2 Abstract and Row 3 Subject terms (Keywords)**
3. Choose **OR** between the rows
4. Select PsycINFO, CINHAL, PsycARTICLES, ERIC, Teachers Reference Centre, Academic Search Premier, and SocIndex)

(“Mental Health” OR “Wellbeing” OR “Emotional Regulation” OR “Resilience” OR “Self-esteem” OR “Coping Skills” OR “Self-regulation” OR “Socio-emotional Characteristics” OR “Socio-emotional Skills”) AND (“Program*” OR “Promotion” OR “Life skills” OR “Socio-emotional Learning” OR “Intervention” OR “Psycho-education”) AND (“Child*” OR “Primary School*” OR “School Child*” OR “Elementary School*”) AND (“School-based” OR “School based” OR “School Setting” OR “Curriculum” OR “School”) AND (“Universal” OR “Group-based” OR “Group based” OR “Classroom-based” OR “Classroom based” OR “Classroom”)

***Missing Records:** If you notice some results are missing, it's often because EBSCOhost **removes exact duplicates** from the final results list before export, especially in the large batch export. The final count in the exported file may be lower than your initial search result count.

## SCOPUS (EMBASE): 2,048 documents found (CL-H & CR)

<http://ezproxy.uct.ac.za/login?url=http://www.scopus.com>

<https://www-scopus-com.ezproxy.uct.ac.za/pages/user/exports> (Specific for exports)

1. Using the advanced search option, Paste EACH search string TERM (e.g. Mental Health) separately into a search row and select Title/Abstract/Keywords
2. Select **Title/Abstract/Keywords for each row**
3. Choose **AND** between the rows

(“Mental Health” OR “Wellbeing” OR “Emotional Regulation” OR “Resilience” OR “Self-esteem” OR “Coping Skills” OR “Self-regulation” OR “Socio-emotional Characteristics” OR “Socio-emotional Skills”) AND (“Program*” OR “Promotion” OR “Life skills” OR “Socio-emotional Learning” OR “Intervention” OR “Psycho-education”) AND (“Child*” OR “Primary School*” OR “School Child*” OR “Elementary School*”) AND (“School-based” OR “School based” OR “School Setting” OR “Curriculum” OR “School”) AND (“Universal” OR “Group-based” OR “Group based” OR “Classroom-based” OR “Classroom based” OR “Classroom”)

## Grey Literature from Scopus-

## 16 secondary documents added (CL-H & CR)

Manual addition - 23 Secondary documents were found and reviewed and (1 by Dray was included twice because there was a review protocol of the included study) Studies EXCLUDED; on End note: 1 was a **duplicate** –(Fenwick) and (Briones) had 1 record for dissertation and another 1 for abstract only so only the dissertation was added to Endnote, Next 3 were a duplicate of abstract reviews which led to the original review that was included on their part (Adi), with manual Search: 2 excluded because it was already added by Scopus under primary docs (Schmitt, Strengthening….) EXCLUDED SONG (2008) because it was an AI generated citation

## 32 preprints found (CL-H & CR)

56 were reported on Scopus but after manual search these were left out: 1 pre-print was excluded because it was removed off the internet- (Gómez Mejía, L.F. , Relph, N.S. & Owen, M-2025 -SSRN). I excluded feeding programmes or school meals and parenting issues and teacher mental health/mental health literacy/programmes/perspectives and postpartum studies, and child hygiene and surveys, COVID specific research not related to interventions, PTSD/Trauma, impact of poverty studies, child protection, GBV, non-school based/centre-based research before loading onto endnote. One record was a duplicate with 4 records showing.

## CENTRAL 12 Reviews results and 832 Trials = 844 (CL-H & CR)

<https://www.cochranelibrary.com/search>

1. Paste the following search string into the 1 search row and select **Title/Abstract/Keywords**

**Or**

1. Using the advanced search option, Paste EACH search string TERM (e.g. Mental Health) separately into a search row and select **Title/Abstract/Keywords**
2. Choose **AND** between the rows

(“Mental Health” OR “Wellbeing” OR “Emotional Regulation” OR “Resilience” OR “Self-esteem” OR “Coping Skills” OR “Self-regulation” OR “Socio-emotional Characteristics” OR “Socio-emotional Skills”) AND (“Program*” OR “Promotion” OR “Life skills” OR “Socio-emotional Learning” OR “Intervention” OR “Psycho-education”) AND (“Child*” OR “Primary School*” OR “School Child*” OR “Elementary School*”) AND (“School-based” OR “School based” OR “School Setting” OR “Curriculum” OR “School”) AND (“Universal” OR “Group-based” OR “Group based” OR “Classroom-based” OR “Classroom based” OR “Classroom”)

## PROQUEST 880 Results <https://www.proquest.com/results/7363C62CCC83448APQ?accountid=14500>

1. Paste the whole search string into the search query in three separate rows
2. Select **Row 1 Title and Row 2 Abstract and Row 3 Abstract and summary text:**
3. Choose **OR** between the rows
4. Select under Source types; Conference Papers & Proceedings, Dissertations & Theses, Government & Official Publications, Reports, Working Papers
5. Select under Document type; Conference Paper, Conference Proceeding, Dissertation/Thesis, Government & Official Document, Report, Working Paper/Pre-Print

(“Mental Health” OR “Wellbeing” OR “Emotional Regulation” OR “Resilience” OR “Self-esteem” OR “Coping Skills” OR “Self-regulation” OR “Socio-emotional Characteristics” OR “Socio-emotional Skills”) AND (“Program*” OR “Promotion” OR “Life skills” OR “Socio-emotional Learning” OR “Intervention” OR “Psycho-education”) AND (“Child*” OR “Primary School*” OR “School Child*” OR “Elementary School*”) AND (“School-based” OR “School based” OR “School Setting” OR “Curriculum” OR “School”) AND (“Universal” OR “Group-based” OR “Group based” OR “Classroom-based” OR “Classroom based” OR “Classroom”)

# TRIALS

## ICTRP 65 records/results

1. Inputted whole Search string approach
2. Clicked clinical trials in children
3. Clicked search

(“Mental Health” OR “Wellbeing” OR “Emotional Regulation” OR “Resilience” OR “Self-esteem” OR “Coping Skills” OR “Self-regulation” OR “Socio-emotional Characteristics” OR “Socio-emotional Skills”) AND (“Program*” OR “Promotion” OR “Life skills” OR “Socio-emotional Learning” OR “Intervention” OR “Psycho-education”) AND (“Child*” OR “Primary School*” OR “School Child*” OR “Elementary School*”) AND (“School-based” OR “School based” OR “School Setting” OR “Curriculum” OR “School”) AND (“Universal” OR “Group-based” OR “Group based” OR “Classroom-based” OR “Classroom based” OR “Classroom”)

## OSF SEARCH 11 Pre-prints Results

(universal school-based mental health)

<https://osf.io/search?search=universal%20school-based%20mental%20health&tab=1>

12 preprints were found but 2 were **duplicates**

**Duplicates** removed before transferring to Rayyan:

1x OSF preprint

880 x EBSCOhost

3x Scopus Pre-prints
