# Supplementary material for: School-Based Universal Mental Health Prevention Programs for Children Aged 6 to 9 Years in Low- and Middle-Income Countries: Protocol for a Scoping Review
Source: JMIR Res Protoc. 2026 Apr 10;15:e87105. doi: 10.2196/87105 (PMC13068186; doi:10.2196/87105)
Supplement: Multimedia Appendix 2 [file resprot-v15-e87105-s002.docx]

## Appendices

### Appendix I: Data extraction tool

| Author | Publi-  cation  year | Coun-try | Setting | target popu-lation | Sample size | Type of inter-vention  e.g. SEL | Program  adaptations for setting | Program com-ponents | Evi-dence for com-  ponents | Method of delivery And Facilitator type | Duration  of program  and sessions | Outcomes: measures, feasibility, accep-tability | Sustain-  ability  Indi-cators | Research findings | JBI appraisal |
| --- | --- | --- | --- | --- | --- | --- | --- | --- | --- | --- | --- | --- | --- | --- | --- |
|  |  |  |  |  |  |  |  |  |  |  |  |  |  |  |  |
|  |  |  |  |  |  |  |  |  |  |  |  |  |  |  |  |
|  |  |  |  |  |  |  |  |  |  |  |  |  |  |  |  |
|  |  |  |  |  |  |  |  |  |  |  |  |  |  |  |  |
|  |  |  |  |  |  |  |  |  |  |  |  |  |  |  |  |
|  |  |  |  |  |  |  |  |  |  |  |  |  |  |  |  |
|  |  |  |  |  |  |  |  |  |  |  |  |  |  |  |  |
